# Supplementary material for: Chromothripsis during telomere crisis is independent of NHEJ, and consistent with a replicative origin
Source: Genome Res. 2019 May;29(5):737–49. doi: 10.1101/gr.240705.118 (PMC6499312; doi:10.1101/gr.240705.118)
Supplement: Supplemental Material [file supp_gr.240705.118_Supplemental_file_1.zip › contigs/annotated_contigs/DB68/contig.2.DB68_length_440_mean_cov_9.80909090909.docx]

**DB68_length_440_mean_cov_9.80909090909**

```
AGAGACCATCAAACAGGCTTTGTGTGAGCAATAAAGCTGTTTATTTCACCTGGGTGCAGGTGGGCTGAGTCCAAAAAGAGAGTCAGCGA
 >chr1:79954310-79954519 + E=3e-114
AGGGAAATAGGGGTGGGGCCGTTTTATAGGATTTGGGTAGATAAAGGAAAATTACAGTCAAAGGGGGGTGTTCTCTGGCCGGCAGAGTG

GGGGTCACAAGGTACTCAGTGGGGGAGCT|TT|ATAAAACAAGACAAAAAAAATTCACTTCCTTCAAACAAGTTACAGATTGTATTACC
                              >chr1:80101353-80101587 + E=2e-124
AACTTAAATTTTGTCCTGTGCAATAGCCTTTCTTATTCTGAAAACAAGCTGACATTTTATTTTAGGGCAAAATGACTTTTTCTTTATCC

CCCAAGGTGGAGAAAGAGGAGGAGACAGGGAAGAGAGGAACACATTCATTACTTTCAGTTGTATTTTGTTGCCACTATCATTCCTA
```
